# A Medical Causerie

**Published:** 1916-05-20

**Authors:** 


### A MEDICAL CAUSERIE.

Many dental surgeons think that their branch of the profession has been very inefficiently utilised by the Army, and the work done at one of the hospitals near London for patients who have received bullet wounds or other injuries to the jaws seems to justify this complaint. The practice of dentistry has suffered prejudice from some of its associations, but there is no doubt that in the surgery of the jaws there is special need for the help of the most skilled dental practitioners, and though this necessity has been contested, it is gradually receiving recognition.

There is widespread dissatisfaction among general practitioners with the decision to reduce the fee for notification of cases of infectious disease from half a crown to one shilling. A medical deputation waited on the Parliamentary Secretary of the Local Government Board, but their protest was unavailing and the decision has now been approved by Parliament. Whether it can be revoked after the war time alone can tell, but in the meantime it stands as the law of the land. The deputation was not altogether happy in suggesting as a compromise that the reduction should be applied to measles but not to other diseases, for this provoked the retort that if a shilling was an adequate fee for one disease it must equally be adequate for others. At the same time it is only fair to recognise that the proposal of the deputation was not advanced on its merits, but was offered as a "contribution to retrenchment" during the period of the war, and in view of the fact that measles has only recently been made notifiable and that the number of cases would probably be large. Seeing that the practitioner has to pay postage, has very often to telephone for an ambulance, and to acknowledge the payment when received, the reduced fee is indeed ridiculous. The responsibility is a considerable one, and the new arrangement will hardly make for smooth working between practitioners and the local health office. Hitherto the latter has been able to depend on the good will and much gratuitous co-operation from those engaged in general practice, but this attitude will not be easily maintained when a sense of injustice is widely felt. The profession has not been more fortunate in its opposition to the proposed abolition of reports from factory surgeons. The Home Secretary has accepted the view that these reports are not essential, and so has decided to make a summary end of them. In regard to the increased duty on motor-cars the doctor will continue to enjoy a reduction of 50 per cent., but he will not escape the new duties altogether.

One effect of the war on medical interests has been the almost complete suspension of post-graduation schools. Some of these have managed to keep their doors open, but teachers and students are equally hard to find. The home practitioners are in the Army or are overwhelmed with private work, and much the same is true of the Colonials. The London School of Tropical Medicine has in this respect shared the fate of its fellows, and for the present can only carry on a difficult existence. But whatever may happen in other directions, this school is sure of a re-established prosperity as soon as we get back to normal times. The new Dean, Sir Richard Havelock Charles, G.C.V.O., will bring to his task both knowledge and energy.

A deputation from Glasgow, which included the Lord Provost and Sir Wm. Macewen, was recently received by Sir Alfred Keogh for the purpose of discussing the arrangements for a hospital for limbless sailors and soldiers proposed to be established in the West of Scotland. One of the purposes of the deputation was to gain the assurance that the new hospital should enjoy the same privileges with regard to grants, etc., and should receive the same recognition from the naval and military authorities, as Queen Mary's Auxiliary Hospital at Roehampton. Indeed, it was bluntly stated that without this assurance the movement in support of the Scottish hospital would not be continued. The request was very readily granted, and, the deputation being satisfied on these points, afterwards paid a visit to Roehampton and thus had the advantage of an introduction to the methods there practised. The need for a further institution is beyond question, for there is a waiting list at the Roehampton Hospital of some 1,500 cases. The new scheme has received substantial support in the West of Scotland, and its success is certain.

Medical societies in this country usually steer clear of political matters, but on the other side of the Atlantic the limits of discussion are less rigidly fixed. Thus the Associated Physicians of Long Island have come to the conclusion that the European war means that treaties between nations are of no binding value, and that any weak and undefended country may become the prey of a more powerful one. From this conclusion they proceed to advocate compulsory military service, or militia training of the type now in force in Switzerland, for every American citizen; a material increase in the standing army and in the navy; the direct association of every one of their members with some organisation active in matters of national defence; the training of medical students in military medicine and surgery, and in the principle of army organisation; and the advocacy of these decisions by medical practitioners among their patients. Such proposals introduced into any of our traditional societies would probably be regarded as out of place; yet under the pressure of the war we are beginning to see that the various departments of national life cannot be shut up in water-tight compartments. Our fighting ranks to-day are formed by men who yesterday never dreamed of being soldiers; and our R.A.M.C. consists mainly of men whose experience has been gained in civil practice. After the war we shall not easily go back to isolation and detachment.

